# Minutes of the Tenth Annual Meeting of the Ohio Dental College Association

**Published:** 1864-03

**Authors:** 


### Proceedings of Societies.

### MINUTES OF THE TENTH ANNUAL MEETING OF THE OHIO DENTAL COLLEGE ASSOCIATION.

The Association met in the lecture room in the Ohio Dental College, February 19th, 1864, Dr. G. W. Keely, first Vice President, in the Chair.

The following members were preseut: Drs. Geo. Watt, A. S. Talbert, S. Driggs, H. A. Smith, H. R. Smith, H. J. McKellops, James Taylor, A. Berry, J. M. Brown, James Leslie, J. Taft, A. M. Leslie, B. D. Wheeler, W. H. Shadoan, J. P. Ulrey, E. Collins.

The minutes of the last meeting were read.

The Committee appointed at the last meeting for procuring stock, was called upon, when Dr. Taylor made some remarks, showing what had been done; he also presented the subscriptions obtained within the last year. They are as follows, viz:

| NAMES. | NO. OF SHARES. |
|---|---|
| C. W. Spalding, | Two Shares. |
| Isaiah Forbes | Two Shares. |
| H. J. McKellops, | Two Shares. |
| Aaron Blake, | Two Shares. |
| Henry Barron, | Two Shares. |
| A. D. Sloan, | Two Shares. |
| A. M. Leslie, | One Share. |
| Geo. H. Cushing, | One Share. |
| W. W. Allport, | Two Shares. |
| J. D. Quinlan, | One Share. |
| J. A. Kennicott, | One Share. |
| Jno. C. Fuller, | One Share. |
| Emanuel Honsinger, | One Share. |
| T. P. Abell, | Two Shares. |
| Wm. Albaugh, | One Share. |
| James C. Dean, | One Share. |
| James Leslie, | One Share. |
| B. D. Wheeler. | One Share. |
| W. P. Horton, | One Share. |
| Alexandria B. Halliwell, | One Share. |
| J. G. Cameron, | One Share. |
| Wm. N. Morrison, | One Share. |
| T. Wrightson, | One Share. |
| Julius Cheesebrough, | One Share. |
| W. H. Shadoan, | One Share. |
| Geo. F. Foote, | One Share. |
| Geo. E. Jones, | One Share. |
| J. Taft, | One Share. |
| Jas. Taylor, | One Share. |
| H. A. Smith, | One Share. |
| H. R. Smith, | One Share. |

Reports were also read by Drs. Taft, Driggs, and Collins. These reports exhibited the fact, that stock sufficient to liquidate the entire indebtedness of the College, has been subscribed, and will be paid within the present year.

On motion of Dr. Watt,

*Resolved,* That the stock of the Ohio Dental College Association, hereafter be a non-interest bearing stock.

The following persons having taken stock since the last meeting, were elected members of the Association, viz:

Dr. Isaiah Forbes, St. Louis, Missouri.
Dr. Aaron Blake, St. Louis, Missouri.
Dr. Henry Barron, St. Louis, Missouri.
Dr. A. D. Sloan, St. Louis, Missouri.
Dr. A. M. Leslie, St. Louis Missouri.

Dr. Geo. H. Cushing, Chicago, Ills.
Dr. J. D. Quinlan, Chicago, Ills.
Dr. J. A. Kennicott, Chicago, Ills.
Dr. Jno. C. Fuller, Chicago, Ills.
Dr. Emanuel Honsinger, Chicago, Ills.
Dr. T. P. Abell, Chicago, Illinois.
Dr. Wm. Albaugh, Chicago, Ills.
Dr. James C. Dean, Chicago, Ills.
James Leslie, Cincinnati, Ohio.
Dr. B. D. Wheeler, Cincinnati, Ohio.
Dr. W. P. Horton, Cleveland, Ohio.
Dr. Alexander B. Halliwell, M. D., Cleveland, O.
Dr. J. G. Cameron, Cincinnati, O.
Dr. Wm. N. Morrison, St. Louis, Mo.
T. Wrightson, Cincinnati, O.
Dr. Julius Cheesebrough, Toledo, O.
Dr. W. H. Shadoan, Cincinnati, O.
Dr. Geo. F. Foote, Cincinnati, O.
Dr. Geo; E. Jones, Cincinnati, O.

On motion of Dr. H. A. Smith,

*Resolved*, That the Stockholders of the College Association, authorize an appropriation sufficient to cover the *actual expenses* incurred by the parties, who have been instrumental in procuring new stock to the College Association.

On motion,

*Resolved*, That the proper officers be authorized to have prepared certificates of stock, in accordance with the above plan, and the resolution just adopted; and that they issue to the Stockholders new certificates, in place of the old ones now out, and to the new subscribers in accordance with the amount subscribed.

On motion,

*Resolved*, That a vote of thanks be hereby tendered to the brethren of the profession, who have so kindly and promptly come forward to our assistance, in liquidating the debt of this institution.

*Resolved*, That the Association now proceed to the election of officers for the ensuing year:

Whereupon the following officers were found to be elected, viz:

*President*, Dr. Chas. Bonsall.

*First Vice President*, Dr. Geo. Watt.

*Second Vice President*, C. W. Spalding.

*Secretary*, J. Taft.

*Treasurer*, J. Taylor.

The President appointed Drs. H. A. Smith and A. M. Leslie, an auditing committee to examine the Treasurer's accounts and report to the Association to-morrow morning.

On motion of Dr. Watt,

*Resolved*, That a committee of two be appointed to nominate delegates to the American Dental Association, to report to-morrow morning.

Committee, Geo. Watt, and J. Taft.

Adjourned to meet to-morrow at 9 A. M.

FEBRUARY 20TH.

The Association met according to adjournment.

The minutes of the former session were and approved.

The Committee to nominate Delegates to the American Dental Association, presented the following names, which were elected, viz:

Dr. A. S. Talbert, Lexington, Ky.

Dr. J. P. Ulery, Rising Sun, Indiana.

Dr. Geo. W. Keely, Oxford, Ohio.

Dr. E. Collins, Cincinnati.

Dr. A. Berry, Cincinnati.

Dr. B. D. Wheeler, Cincinnati.

Dr. M. DeCamp, Mansfield, Ohio.

Dr. J. C. Dean, Chicago, Illinois.

Dr. H. E. Peebles, St. Louis, Missouri.

Dr. I. Forbes, St. Louis, Missouri.

Dr. H. Barron, St. Louis, Missouri.

Dr. A. M. Leslie, St. Louis, Missouri.

Dr. T. P. Abell, Chicago, Illinois.

Dr. W. P. Horton, Cleveland Ohio.

The auditing committee made the fellowing report:

REPORT OF THE TREASURER FOR 1863.

CINCINNATI, February 20th, 1863.

The Treasurer would respectfully submit the following report, embracing the assets of the Association for the year just preceding the last meeting.

The interest on the mortgage has all been paid up to July 1st, 1863. The necessary repairs of the Institution have been attended to and bills paid up.

The building is kept insured to the amount of five thousand dollars, and the policy paid up to September next.

The Treasurer has received from the Faculty $435,00 which have been appropriated as follows: interest on mortgages, $400,00, Insurance, $35,00.

JAS. TAYLOR, *Treas.*

We have examined the Treasurers accounts and find them correct.
A. M. LESLIE,
H. A. SMITH.

REPORT OF TREASURER FOR 1864.

CINCINNATI, February 20th 1864.

The Treasurer would respectfullyy submit the following report for the account current for the year ending this day:

| | | |
|---|---|---|
| Received from Facul·y | | $435 00 |
| Paid on Interest of Mortgage | $400 00 | |
| Paid on Insurance of Building | 35 00 | —435 00 |

The Treasurer would also respectfully submit the following, viz: the deficit reported Feb. 20, 1862, of $355,39, has been reduced by the receipt of the following sums:

| | | | | | |
|---|---|---|---|---|---|
| March 3d, 1862, | Cash of J. M. Lewis | on share of | Stock | $ | 20 00 |
| June 10, | " Cash of W. W. Allport, | " | " | | 50 00 |
| " 10 | " " Eli Collins, | " | " | | 50 00 |
| Feb. 20, 1863, | " " | " | " | | 50 00 |
| Feb. 20, | " " W. W. Allport, | " | " | | 50 00 |
| Feb. 20, | " " Dr. Driggs, | | | | 100 00 |
| | | | | | $320 00 |

Leaving a balance of thirty-five dollars and and thirty-nine cents on floating debt to be liquidated.

Respectfully submitted,
JAS. TAYLOR, Treasurer.

We have examined the Treasurers accounts and find them correct. H. A. Smith,
A. M. Leslie.

At this point quite a spirited discussion arose in reference to the furnishing of the Laboratory and Infirmary, pending which, on motion of Dr. McKellops,

*Resolved*, That the Faculty appoint a committee of one, in each city and town in which there may be stockholders, to solicit contributions in money, appliances, fixtures, books, &c., for the purpose of placing these departments in the best possible condition.

(A beginning was made in this direction there in the meeting, by a subscription of $250,00 part of which was paid in.)

After some farther discussion and consultation, in which the liveliest interest was manifested on behalf of the Institution, the Association adjourned to meet February 18th, 1865.

